# A Comparison of Retinyl Palmitate and Red Palm Oil β-Carotene as Strategies to Address Vitamin A Deficiency

**DOI:** 10.3390/nu5083257

**Published:** 2013-08-15

**Authors:** Ellie Souganidis, Arnaud Laillou, Magali Leyvraz, Regina Moench-Pfanner

**Affiliations:** 1 Johns Hopkins School of Medicine, Baltimore, MD 21287, USA; E-Mail: esougan1@jhmi.edu; 2 University of Montpellier II, Science and Technology, Montpellier 34000, France; 3 Global Alliance for Improved Nutrition, Geneva 1200, Switzerland; E-Mails: mleyvraz@gainhealth.org (M.L.); rmoenchpfanner@gainhealth.org (R.M.-P.)

**Keywords:** oil, fortification, retinyl palmitate, beta-carotene, red palm oil, Vitamin A

## Abstract

Vitamin A deficiency continues to be an international public health problem with several important health consequences including blindness and overall increased rates of morbidity and mortality. To address this widespread issue, a series of strategies have been put into place from dietary diversification to supplementation and fortification programs. Retinyl palmitate has been used successfully for decades as a supplement as well as a way to fortify numerous foods, including vegetable oil, rice, monosodium glutamate, cereal flours and sugar. Recently, there has been rising interest in using a natural source of carotenoids, β-carotene from red palm oil (RPO), for fortification. Although RPO interventions have also been shown to effectively prevent Vitamin A deficiency, there are numerous challenges in using beta-carotene from RPO as a fortification technique. β-Carotene can induce significant changes in appearance and taste of the fortified product. Moreover, costs of fortifying with beta-carotene are higher than with retinyl palmitate. Therefore, RPO should only be used as a source of Vitamin A if it is produced and used in its crude form and regularly consumed without frying. Furthermore, refined RPO should be fortified with retinyl palmitate, not β-carotene, to ensure that there is adequate Vitamin A content.

## 1. Introduction

Vitamin A deficiency affects approximately 125–130 million preschool-aged children and 7 million pregnant women in low-income countries [1] and is the leading cause of preventable pediatric blindness in the developing world [2]. It also increases susceptibility to severe infections [3] and results in an elevated risk of mortality in children and pregnant and lactating women [4,5]. The most common cause of Vitamin A deficiency is insufficient dietary intake of Vitamin A, which is normally found in animal source foods as preformed Vitamin A (or retinol) and in plant source foods as Provitamin A. In developing countries, the widespread consumption of primarily vegetable-based diets has exacerbated Vitamin A deficiency because of the poor bioavailability of Provitamin A carotenoids [6,7]. Although considerable progress has been made in controlling Vitamin A deficiency worldwide [8], there is still a need for additional prevention efforts in the form of dietary diversification, fortification, and supplementation [9].

Fortification programs have been shown repeatedly to be an effective food-based strategy to improve Vitamin A status [10,11]. While fortification of vegetable oil with Vitamin A is considered one of the most cost-effective and easily implementable strategies [12], retinyl palmitate, the most commonly used Vitamin A fortificant, has also been used successfully to fortify sugar, monosodium glutamate, and wheat flour [13,14,15,16,17,18]. In red palm oil (RPO) producing countries, some stakeholders are questioning whether the fortification of vegetable oil with β-carotene from RPO can be as effective as fortification with retinyl palmitate given its Provitamin A activity and high bioavailability [19,20]. Despite the high concentration of carotenoids in RPO, questions have arisen regarding the health implications of increased β-carotene consumption, the stability of RPO during food preparation, and the overall feasibility of RPO as a fortification strategy.

The purpose of this paper is to review the scientific literature on the feasibility of RPO β-carotene fortification as a strategy to address Vitamin A deficiency and to compare this approach to previous efforts using retinyl palmitate. Evidence from previously performed stability and fortification tests will form the basis of the review. Additional information will be drawn from the medical literature on the health implications of beta-carotene. Ultimately, we predict that there is evidence to support the use of crude RPO as a source of Vitamin A without the need for additional fortificant. However, refined RPO should be fortified with retinyl palmitate to restore the Vitamin A content of the oil that is lost through processing.

## 2. The Evolution of Vitamin A Fortification through Retinyl Palmitate and RPO

Vitamin A’s high fat-solubility has greatly facilitated its role as a fortificant, particularly in fat-based or oily foods. Although preformed Vitamin A is highly unstable, esterification techniques using palmitic and acetic acid have yielded more stable esters in the form of retinyl palmitate and retinyl acetate, respectively [21]. Recommendations by the World Health Organization to combat Vitamin A deficiency and xerophthalmia include prevention strategies based on oral administration of retinyl palmitate [22,23]. In an effort to develop new approaches for reducing the prevalence of Vitamin A deficiency, retinyl palmitate has also become the most commonly used Vitamin A food fortificant [24] and has been used to fortify a variety of foods based on geographical location and local availability [21].

Oil is the most suitable vehicle for Vitamin A fortification and efforts to fortify vegetable oil with retinyl palmitate have been well-established at a low cost. The oil matrix protects against the oxidation of Vitamin A during storage, improves stability of the retinol, and facilitates the vitamin’s absorption by the body [25,26]. The advantages of oil fortified with retinyl palmitate have historically been utilized by food aid programs, including the U.S. Title II Food Aid Program (U.S.PL480), where a daily intake of 16 g of oil provided approximately 50% of the recommended daily intake (RDI) of an adult male [27]. Other successful methods of food fortification with retinyl palmitate have included margarine, cereal products and flours, and sugars, and have provided the basis for country-based programs. In India, for example, vanaspati (a hydrogenated oil) has been fortified with retinyl palmitate since 1953 [28] and retinyl palmitate fortification of margarine in Newfoundland, Canada has yielded significant improvements in the population’s Vitamin A status since the program’s initiation in 1944–1945 [29]. Despite widespread use of retinyl palmitate, additional strategies for Vitamin A food fortification have emerged and in the case of RPO, have reflected geographic variability and regional preferences.

Crude RPO is a natural source of carotenoids, tocopherols, and tocotrienols, all of which contribute to the stability and nutritional properties of the oil [30]. Carotenoids, particularly α- and β-carotene, are responsible for the orange-red color of crude RPO and provide oxidative protection [31]. Tocopherols and tocotrienols, both isomers of Vitamin E, also contribute to the oxidative stability of the oil. Crude RPO is traditionally used for cooking in West and Central African countries. The intense red color and unique taste make crude RPO unfavorable in other regions of the world, prompting the development of palm oil processing techniques, particularly in Malaysia, to produce nearly colorless refined oil. Crude RPO may undergo either physical or chemical refining, both processes involving high-temperature deodorization and deacidification under vacuum [32]. The processing removes some of the tocopherols and tocotrienols and destroys all carotenoids present in the crude RPO [33]. More recently, a modified physical refining process has been developed and patented by the Palm Oil Research Institute of Malaysia (PORIM) [34,35]. The softly refined RPO retains 80% of the original carotenoid content but as a result, also retains the unfavorable red color originally present in the crude RPO [36].

## 3. Comparing the Stability of Retinyl Palmitate and β-Carotene

The stability of retinyl palmitate as a food fortificant has been assessed in a variety of oil products. In a 1991 study, Favaro and colleagues found that 99% of the Vitamin A content in retinyl palmitate fortified soybean oil is maintained following 9 months of storage at 23 °C [17]. Retention rates of retinyl palmitate were 88% and 90% following 90 min of boiling and 40 min of cooking under pressure, respectively; however, repeated cycles of frying and storage resulted in a progressive loss of Vitamin A content depending on the number of times the oil was reused [17]. Despite this decline, more than 58% of Vitamin A content remained following four pan-fryings of potatoes at 115–117 °C, demonstrating the overall stability of retinyl palmitate in oil with a variety of food preparation techniques [17,37]. In a more recent study, there was a significant amount of retinyl palmitate retained following 30 h of frying at 185 °C in palm, corn, and soybean oil, with the highest retention rate in palm oil [18]. Although temperature stability continues to be the primary focus of oil fortification studies, it is also important to consider the effects of peroxide and acid on fortified vegetable oil stability, especially in light of industry and government regulations. For example, one study published in 2012 found a significant increase in retinyl palmitate decay once the peroxide levels in oil were increased to more than 2 mEq of active oxygen per gram [38]. Of the many countries that have started to fortify vegetable oils with Vitamin A within the last decade, most have kept a standard peroxide value of less than 10 mEq/g for fortified vegetable oil [38]. Therefore, it is recommended to consider adjusting the peroxide and acid values for fortified vegetable oil to fit national standards and preserve retinyl palmitate content.

Ultra Rice (UR), first produced from broken rice grains bound to Vitamin A and other fortificants in the late 1980’s [39], has also been a focus of scientific studies on the utility of retinyl palmitate as a food fortificant. In stability studies, 75 to 87% of retinyl palmitate content in UR was retained, depending on the cooking technique [40]. These findings were consistent with other studies demonstrating a high retention rate of retinyl palmitate in rice following boiling or heating at a low temperature [41,42,43]. After a 6 month storage period, retinyl palmitate in UR was shown to be more affected by temperature than by humidity, with an 85% retention of retinyl palmitate in UR stored at 23 °C compared to significant loses of retinyl palmitate content at temperatures over 35 °C [40].

Potential changes in retinyl palmitate content secondary to long-term storage and temperature variations continue to drive stability studies in other food products. In a study measuring the stability of retinyl palmitate-fortified corn flakes, 90% of the retinyl palmitate content was lost following 6 to 8 weeks of storage, except in samples of cereals fortified with a complete vitamin mixture and kept at room temperature [44]. The high levels of retinyl palmitate degradation were potentially attributed to the corn flakes’ large surface area and a decreased use of antioxidants such as tocopheral or BHT/BHA combinations as compared to the previous studies involving fortified rice [42,44]. Differences in retention rates of retinyl palmitate following comparable food preparation techniques have also been observed in studies involving extrusion-cooking processing, further demonstrating the effect of different food preparation techniques and the value of carefully selecting a fortification vehicle that may promote stability of retinyl palmitate [45,46].

More recently, the stability of retinyl palmitate in microcapsules containing other micronutrients has also been investigated. Spray cooling, also known as “matrix” encapsulation, has been used to generate microcapsules containing retinyl palmitate, iron, and iodine [47]. Although there was a significant loss of retinyl palmitate during production, only 12% of the retinyl palmitate content was lost during 6 months of storage; this observation was attributed to the microcapsule’s hydrogenated fat content, which served as a barrier to oxygen [47]. Overall, stability studies have reinforced the utility of retinyl palmitate as a reliable fortification strategy for Vitamin A with the highest retention rates observed in fortified oils and rice products.

The possibility of fortifying vegetable oil with β-carotene has prompted similar investigations into the stability of the Vitamin A content using different food processing and preparation techniques. In one *in vitro* study, an increased rate of trans-β-carotene degradation was observed in both palm olein and vegetaline following an initial increase in temperature. After 30 min at a temperature of 140 °C, 20% and 50% of trans-β-carotene remained in palm olein and Vegetaline, respectively, whereas less than 10% remained in both oils following treatment at 180 °C [48]. The adverse effects of increased temperature on β-carotene content were reinforced using samples of Nigerian palm oil, which were heated at temperatures ranging from 138 °C to 258 °C. An even greater decrease in β-carotene was observed with increased temperatures or following 30 minutes of continuous heating at any temperature [49].

Although laboratory studies have investigated the stability of β-carotene under controlled conditions, attempts to incorporate β-carotene into food products have presented additional challenges due to its unfavorable color, flavor, and aroma. In addition, repeated processing markedly reduces the antioxidant properties of β-carotene, jeopardizing the stability of the pigment under normal storage conditions. A 1993 US Patent found the incorporation of β-carotene into cereal products, either in the form of water insoluble beadlets or in a finely divided particulate form, to be an effective source of Vitamin A without causing any significant changes in color, aroma, or flavor and with minimal risks of oxidation [50]. Two additional studies measured the effect of baking on the stability of β-carotene, and found a 20 to 30% loss in bagels and cookies [51], a 4%–15% loss in wheat bread, and an 18%–23% loss in crackers, with improvements in stability observed with antioxidant use [52]. While these studies investigate the potential of β-carotene fortification in grain products, they do not address cooking oil as a vehicle for fortification or the effect of other food preparation methods on β-carotene stability.

Numerous studies with RPO have shown that only a fraction of β-carotene is retained when the oil is used for frying [48,49,53]. Degradation of *trans*-β-carotene into 13-*cis*- and 9-*cis*-β-carotene causes the oil to change into dark grey and black colors, respectively [54]. A similar phenomenon has also been observed when refined soybean oil, manufactured and consumed in Brazil, is fortified with *trans*-β-carotene prior to its use as cooking oil; although 92% of β-carotene is retained after cooking at 100 °C for 20 min, there is only 65% retention rate following 3 min of frying at 170 °C [55]. Overall, research on the stability of RPO-derived β-carotene largely focusses on the damaging effects of frying on β-carotene content of cooking oil. As a result, other interventions have focused on fortification techniques that do not involve frying or rely on milder cooking approaches, as well as supplementation.

## 4. Evaluation of Retinyl Palmitate and RPO-Based Nutritional Interventions

Studies comparing the utility of RPO in improving Vitamin A status to synthetic sources of Vitamin A, such as retinyl palmitate, date back to the 1960’s. In 1963, Roels and colleagues compared the effect of daily supplementation of 1 gram of RPO/kg of body weight to 2000 I.U. Vitamin A acetate in 52 Indonesian male children. Serum retinol levels rose and remained elevated among individuals in both groups and serum carotene levels rose dramatically in those who received RPO [56]. A larger community-based trial study 4 years later also found improved serum retinol concentrations after one year in groups receiving either daily doses of 4 mL RPO or 2400 I.U. of synthetic Vitamin A [57]. However, a more recent study from 2002, found that RPO groups experienced a greater increase in retinol and β-carotene levels compared to groups receiving ground nut oil fortified with retinyl palmitate [58]. While the number of studies directly comparing the effects of retinyl palmitate and RPO on Vitamin A status is limited, the existing studies demonstrate that RPO may be as effective as retinyl palmitate when added to the diet in a way that preserves the original contents of the oil. Additional studies have focused specifically on RPO and further reinforced its potential as a nutritional intervention to improve Vitamin A status.

Studies investigating the effects of RPO-incorporated diets have primarily focused on children and pregnant or lactating women, two high-risk populations for Vitamin A deficiency. Two RPO supplementary feeding trials in India found a decreased prevalence of Bitot’s spots in preschool children following the daily incorporation of RPO into meals [59,60]; a similar reduction in Vitamin A deficiency was observed in primary school-aged children in Burkina Faso following 15 mL RPO supplementation three times per week [61]. A recent study in Indonesian children investigated the effects of daily consumption of capsules containing β-carotene dissolved in oil. Although the conversion of β-carotene to retinol was 27% more effective than previous estimates, the intervention’s bioefficacy was only 39% [62]. Furthermore, implementation of the Indonesian model would provide additional challenges by adding a cost and a color to vegetable oil that is not suitable at the consumer level.

As Vitamin A deficiency is also a significant problem in pregnant and lactating women, a 2001 study with pregnant Tanzanian women tested the effects of supplemental RPO and sunflower oil on maternal Vitamin A status from the third trimester to 3 months postpartum. The RPO group showed significant increases in both plasma and breast-milk retinol concentrations with no decline in breast-milk retinol concentrations from 1 to 3 months postpartum [63]. This study confirmed the results from an earlier study in India, where pregnant women and their newborn infants had higher serum retinol concentrations than the control group following a RPO intervention [64]. While this study promoted the use of RPO for food preparation, the women were advised to add the oil towards the end of the cooking process and not to use the oil for frying [63]. Overall, compliance with oil consumption was high, with only one woman declining to regularly consume the oil as requested and two cases of over- or under-consumption, which were immediately addressed [64]. These results indicate that although RPO use can be potentially beneficial in certain methods of food preparation, it may not be correct to assume that individuals would change their food preparation techniques to fit these standards, especially without education on the benefits of using the oil. Furthermore, initiating changes in food preparation techniques would require social marketing campaigns in addition to education to promote and sustain such individualized lifestyle changes.

Given the ongoing success of crude RPO has a strategy to improve Vitamin A status, RPO has also been trialed as a dietary diversification strategy in non-consuming areas of the world. In Burkina Faso, for example, mothers and school-children were the target of a study that look at changes in serum retinol levels once RPO was introduced into the diet. Social marketing strategies were implemented [65] and after 24 months, nearly 45% of mothers and children reported consuming RPO within the last week [66]. Furthermore, the prevalence of low serum retinol levels (<70 µmol/L) decreased from 61.8 ± 8.0% to 28.2 ± 11.0% in mothers and from 84.5 ± 6.4% to 66.9 ± 11.2% in children [65]. Despite the improvement in serum retinol concentrations in both mothers and children compared to baseline, the prevalence of low serum retinol concentrations remained high, particularly in children. Furthermore, the Burkina Faso studies raised concerns regarding the sustainability of RPO programs, particularly with regards to cost and the need for commercial retail channels to produce, store, and market RPO containing adequate levels of β-carotene.

## 5. The Cost of Retinyl Palmitate or β-Carotene Fortification in Vegetable Oil

Vitamin A palmitate 1.7 Mio IU/g is the most widely used fortificant worldwide and appears to be a cost-effective form of Vitamin A for fortification of edible oil as it costs approximately 57.5 USD/kg (ex-factory) according to the GAIN premix facility [67]. Therefore if countries wanted to fortify their vegetable at a level of 45 IU/g, the addition of palmitate would add a fee of 1.52 USD/MT. Despite the low cost of such fortification, many RPO-producing countries continue to question whether β-carotene should still be used because of its availability. The carotene, which is derived from a palm oil through saponification, drying and solvent extraction, is traditionally used as a coloring agent for a wide range of foods such as margarine, beverage, sauces, *etc.* Therefore due to its high demand but also due to the use of RPO as a biofuel, β-carotene costs approximately 130 USD per kilogram (at a concentration of 30 µg of Retinol Equivalent in 1mg of substance) [67]. By using a conversion factor of 12 to transform β-carotene to a retinol equivalent, the amount of β-carotene that would be required to obtain the same fortification level than before (45 IU/g) would cost 65 USD/MT, with on-going concerns regarding the color of the product.

## 6. Health Implications of Retinyl Palmitate and β-Carotene

Vitamin A has been recognized for a long time as an essential micronutrient for health. However, regular overconsumption of Vitamin A can have negative effects on health, including vomiting [68], liver damage [69], bone demineralization and fractures [70,71], birth defects [72,73], and dermatitis [74]. The tolerable upper intake level of Vitamin A for daily doses is estimated to be 900 µg RE (3000 IU) for infants and 7500 µg RE (25,000 IU) for children and pregnant women [75]. Although β-carotene is less of a concern in terms of potential Vitamin A toxicity given its regulated conversion to vitamin A and decreased absorbability at high doses, numerous studies have investigated the health implications of β-carotene, particularly cancer and cardiovascular disease. In lipid phases, β-carotene efficiently reduces peroxyl radicals [76] and quenches ^1^O_2_ [77]. While these antioxidant properties provide the foundation for a number of epidemiologic studies supporting β-carotene’s potential role in cancer prevention [78], results have largely been inconclusive. Instead, the effects of β-carotene appear to be inconsistent between cancer type and population characteristics, with β-carotene levels having been associated with variable effects on carcinogenesis in different sites in the body [78].

Investigations into the potential role of β-carotene have largely focused on gastric carcinomas. A 3-year-long nutritional intervention study in Venezuela tested the efficacy of daily supplementation with β-carotene (18 mg/day), Vitamin C (750 mg/day), and Vitamin E (600 mg/day) on the chemoprevention of gastric premalignancy. Although there was a noticeable regression of gastric premalignant lesions in the treatment group compared to the controls, the results were not statistically significant and could not be used to support β-carotene’s proposed role as a chemopreventive agent [79]. A study in China, however, concluded that a 5.25 year-long supplementation regimen of β-carotene, α-tocopherol, and selenium could significantly reduce gastric cancer mortality by up 21% in a nutritionally inadequate population with markedly high rates of upper gastrointestinal tract cancers [80]. A Columbian study also reported a decrease in gastric premalignant lesions in a high-risk population following either a β-carotene or ascorbic acid intervention, suggesting a potential strategy to prevent gastric carcinoma [81].

Adverse effects of β-carotene administration have been described in the Alpha Tocopherol Beta-Carotene Trial (ATBC) [82] and the Carotene and Retinol Efficacy Trial (CARET) [83], two randomized primary-prevention trials focused on the risk of developing lung cancer. In the ATBC trial, β-carotene supplementation was associated with an increased risk for lung cancer (relative risk (RR) = 1.16; 95% confidence interval (CI) = (1.02–1.33), *p =* 0.02) with a greater effect in heavy smokers and alcohol users [84]. These results were reinforced by results from the Beta-Carotene and Retinol Efficacy Trial (CARET), which found an elevated risk of lung cancer incidence (RR = 1.36; 95% CI = (1.07–1.73); *p* = 0.01) and lung cancer mortality (RR = 1.59; 95% CI = (1.13–2.23); *p* = 0.01) in the intervention group compared to the placebo group [85]. While both trials demonstrated an increased risk of lung cancer and lung cancer mortality in smokers and other high risk group receiving β-carotene supplementation, the joint administration of 30 mg of β-carotene and 25,000 retinyl palmitate in the CARET treatment group made it unclear as to the cause of these adverse effects [86]. Possible explanations for the elevated relative risk observed in the CARET trial include a higher dose of β-carotene or a potential interaction between β-carotene and retinyl palmitate. Given that retinyl palmitate has been shown to be effective in preventing the development of primary lung tumors associated with tobacco consumption and in increasing the disease-free survival in patients who had previously undergone surgery for removal of a primary lung tumor in patients receiving adjuvant treatment with high dose Vitamin A [87], the authors concluded that it was likely the effect of β-carotene on the development of lung cancer in high risk groups.

The role of β-carotene in cardiovascular disease has also been investigated with similar mixed outcomes. While observational epidemiological studies have provided general support for the role of antioxidants, including β-carotene, in reducing cardiovascular disease [88,89], corroboration from interventional trials is lacking [90]. The MRC/BHF Heart Protection Study and the Physicians Health Study found that β-carotene had no effect on reducing cardiovascular events [91,92] and two additional randomized control trials found a possible increased risk of cardiovascular events [82,93]. The Rotterdam Study, on the other hand, found that high intake of β-carotene was protective against myocardial infarction, with a more pronounced effect in people with a current or past history of smoking [94]. A recent meta-analysis of 8 randomized trials, including the ATBC and CARET studies, evaluated the impact of β-carotene on cardiovascular disease and found β-carotene to result in a minor but statistically significant increase in all-cause mortality (Odds Ratio (OR) = 1.07; 95% CI = (1.02–1.11); *p* = 0.003) and cardiovascular death (OR = 1.1; 95% CI = (1.03–1.17); *p* = 0.003) [95]. However, the results from trials addressing the risks of cancer and cardiovascular disease following increased β-carotene intake have been largely inconsistent as a whole and may reflect the effect of different dosing regimens. Therefore, the risks of β-carotene consumption as part of Vitamin A fortification programs must be interpreted within the context of how much β-carotene is consumed and over what period of time. Additional studies are warranted to compare risks of negative health outcomes between β-carotene and retinyl palmitate over prolonged periods of supplementation or fortification.

## 7. Conclusions

Existing evidence supports the claim that RPO-interventions can be efficacious in preventing Vitamin A deficiency in populations at risk [56,57,58,59,60,61,63,64,66]. However, the feasibility, effectiveness, and sustainability of such programs are dependent on the cost of such interventions, the physical characteristics of RPO, and the types of traditional food preparation strategies used. Therefore, RPO should only be used as a source of Vitamin A if it is produced and used in its crude form and regularly consumed without frying. Furthermore, refined RPO should be fortified with retinyl palmitate, not β-carotene, to ensure that there is adequate Vitamin A content. Ultimately, the use of RPO is highly dependent on geographical location and local preferences and must be carefully monitored to ensure that it is providing the physiologically-appropriate level of Vitamin A to its population.
